# Green-Extracted *Ficus carica* L. Fruit Polysaccharides Promote Longevity in *Caenorhabditis elegans* via Modulation of SKN-1 and IIS Pathway

**DOI:** 10.3390/antiox15060691

**Published:** 2026-05-30

**Authors:** Lianyu Li, Feng Ding, Yong Sheng, Yan Zhao

**Affiliations:** 1 School of Chemistry and Chemical Engineering, Harbin Institute of Technology, Harbin 150001, China; 2 Department of Bioengineering, Harbin Institute of Technology, Weihai 264209, China

**Keywords:** polysaccharides, *Ficus carica* L., lifespan, oxidative stress, skinhead-1 (SKN-1), insulin/IGF-1 signaling (IIS)

## Abstract

In this study, polysaccharides from *Ficus carica* L. fruits (FCPs) were extracted using a deep eutectic solvent (DES)-based ultrasound-assisted extraction (UAE) method. The physicochemical properties of the FCPs were then characterized, and the anti-aging effects of FCPs were evaluated in *Caenorhabditis elegans* (*C. elegans*). It was demonstrated that FCPs significantly extended the lifespan of the nematodes, while improving locomotor activity without affecting the body size or reproductive capacity. Meanwhile, FCPs reduced lipofuscin accumulation, decreased intracellular reactive oxygen species (ROS) levels, and increased the survival of *C. elegans* under oxidative stress. Moreover, FCPs upregulated the expression of antioxidant genes *sod-1*, *sod-3*, *ctl-2*, *ctl-3* and *gst-4*. The expression of *skinhead-1* (*skn-1*), a homologue gene of mammalian *nuclear factor erythroid 2-related factor* (*Nrf*) in *C. elegans*, was also elevated upon FCPs treatment. Knockdown of *skn-1* expression by RNA interference abolished the lifespan extension and ROS reduction in FCPs-treated *C. elegans*, indicating that the SKN-1-mediated signaling was essential for the anti-aging effects of FCPs. Additionally, FCPs caused downregulation of the key components of the insulin/IGF-1 signaling (IIS) pathway, *age-1*, *akt-1*, and *akt-2*. Overall, these results suggested that FCPs promoted longevity in *C. elegans* via modulation of SKN-1 and IIS pathway.

## 1. Introduction

Aging is a complex biological process, and its molecular mechanisms remain at the forefront of scientific research [1]. Reactive oxygen species (ROS) act as signaling molecules that regulate stress-response pathways, adaptive defense, and longevity-related processes [2,3]. However, overproduction of ROS has been implicated as one major event contributing to aging [1]. The first pathway discovered to regulate aging, insulin/insulin-like growth factor-1 (IGF-1) signaling (IIS), is central to many important cellular decisions, ranging from growth, development, metabolism, and reproduction to longevity [4]. Notably, the IIS pathway also modulates antioxidant defenses through negative regulation of transcription factor skinhead-1 (SKN-1) in *Caenorhabditis elegans* (*C. elegans*) [5,6]. SKN-1 is an ortholog of mammalian nuclear factor erythroid 2-related factor (Nrf), which controls the expression of a series of antioxidant genes and is critical for maintaining cellular redox homeostasis [7]. Evidence suggests that SKN-1 plays an important role in the regulation of aging and stress resistance. Overexpression of SKN-1 significantly extends the lifespan of *C. elegans*, whereas the loss of function *skn-1* leads to shortened lifespan and reduced stress tolerance [6].

*Ficus carica* L., a deciduous tree of the Moraceae family, produces the edible fruit known as the fig [8]. For centuries, figs have been valued as a food source that benefits human health [9]. Various bioactive components found in figs, including polysaccharides, polyphenols, flavonoids, triterpenoids, and vitamins, contribute to their pharmacological properties [10]. Fig-derived polysaccharides have been reported to exhibit bioactivities such as antioxidant, antitumor and immunomodulatory effects [9,11,12]. Nevertheless, their activity against aging and their impact on lifespan regulation have not been established.

Deep eutectic solvents (DESs) have emerged as a new class of green and effective solvents for polysaccharide extraction, primarily due to their unique capacity to interact with target molecules through hydrogen bonding, which facilitates efficient dissolution of polysaccharides under relatively mild conditions, thus helping preserve the structural integrity and bioactivity of the extracted polysaccharides [13]. It has been shown that DESs can improve the extraction yield and quality of polysaccharides from fig fruit peel compared with conventional methods [14]. Ultrasound-assisted extraction (UAE), which promotes solvent penetration and enhances extraction efficiency [15,16], has been successfully applied in the extraction of polysaccharides from various plant sources [17]. However, the combination of UAE with DESs for the extraction of polysaccharides from figs has not yet been reported.

The present study aimed to extract bioactive polysaccharides from *Ficus carica* L. fruits using a DES-based UAE strategy. The anti-aging effects of the resulting polysaccharides, named FCPs, as well as the possible underlying mechanisms, were then evaluated using *C. elegans* as a model organism.

## 2. Materials and Methods

### 2.1. Materials and Reagents

The fig fruits were purchased from Weihai, Shandong Province, China. Standard monosaccharides, trifluoroacetic acid (TFA), and 2′,7′-dichloro dihydrofluorescein diacetate (H_2_DCFDA) were obtained from Sigma Chemical (Saint Louis, MO, USA). All chemicals used for DES preparation were of analytical grade and purchased from Sinopharm Chemical Reagent Co., Ltd. (Shanghai, China), unless otherwise stated. The reverse-transcription reaction system was purchased from Invitrogen (Eugene, OR, USA). The qPCR reaction systems were purchased from Sangon Biotech (Shanghai, China).

### 2.2. Preparation and Purification of Ficus carica Fruit Polysaccharides

Before extraction, the *Ficus carica* fruit powder was washed with 95% ethanol at a liquid-to-solid ratio of 5 mL/g for 2 h to remove free sugars, pigments, and other small molecules, and then dried at room temperature. *Ficus carica* fruit polysaccharides were extracted using DES-based UAE. Briefly, different DES systems were screened and choline chloride/1,3-butanediol (ChCl/But) was selected as the extraction solvent according to the polysaccharide yield. Single-factor experiments were then performed to optimize the DES-UAE conditions, and the detailed experimental design was provided in the Supplementary Materials Section S1. The optimized extraction conditions were as follows: liquid-to-solid ratio of 20 mL/g, ultrasonic time of 50 min, water content of 10%, and extraction temperature of 60 °C.

Under the optimized conditions, 10 g of pretreated fig fruit powder was mixed with 200 mL of ChCl/But DES containing 10% water and extracted by ultrasound-assisted extraction at 600 W for 50 min at 60 °C. After extraction, the mixture was centrifuged at 4000× *g* for 15 min, and the supernatant was collected and concentrated. Crude polysaccharides were precipitated by adding four volumes of 95% ethanol and kept at 4 °C for 24 h. The precipitate was collected by centrifugation at 4000× *g* for 15 min and redissolved in deionized water.

The crude polysaccharide solution was dialyzed against deionized water using a dialysis membrane with a molecular weight cut-off of 3500 Da for 48 h, and the dialysis water was replaced every 4 h. The retentate was purified using a D101 macroporous resin column (3.5 × 50 cm), which was eluted with distilled water at a flow rate of 2 mL/min. The carbohydrate-containing fractions were collected, concentrated under reduced pressure, and lyophilized using a freeze dryer at −60 °C for 24 h. The obtained purified fig polysaccharide fraction was designated as FCPs.

### 2.3. Chemical Composition, Molecular-Weight Distribution, and Monosaccharide Composition

The total carbohydrate percentage of the FCPs was determined using the phenol-sulfuric acid method with glucose as the standard [18]. The reducing sugar content was quantified using the dinitrosalicylic acid method [19]. Protein content was determined using the Bradford method [20]. Total phenolic content was determined using the Folin–Ciocalteu colorimetric method [21]. The uronic acid was measured using the m-hydroxybiphenyl method [22].

The apparent molecular-weight distribution of FCPs was determined by high-performance gel permeation chromatography coupled with differential refractive index detection (HPGPC-dRI). Briefly, FCPs were dissolved in 0.1 mol/L NaNO_3_ aqueous solution at a final concentration of 10 mg/mL and filtered through a 0.45 μm membrane before analysis. The chromatographic system consisted of a Waters 1515 chromatographic pump and a Waters 2707 autosampler (Waters Corporation, Milford, MA, USA), equipped with a differential refractive index detector (Optilab REX, Wyatt Technology, Goleta, CA, USA). Separation was performed using two serially connected gel permeation columns, Ohpak SB-805 HQ (300 × 8 mm) (Shodex, Tokyo, Japan) and Ohpak SB-803 HQ (300 × 8 mm) (Shodex, Tokyo, Japan). The mobile phase was 0.1 mol/L NaNO_3_ aqueous solution, and the column temperature was maintained at 45 °C. The injection volume was 50 μL, and the total analysis time was 35 min. Data acquisition and analysis were performed using ASTRA software version 6.0 (Wyatt Technology, Goleta, CA, USA). The apparent molecular weight of FCPs was estimated based on calibration with dextran standards.

Monosaccharide composition of FCPs was analyzed by 1-phenyl-3-methyl-5-pyrazolone (PMP) pre-column derivatization followed by high-performance liquid chromatography (HPLC). Briefly, 2 mg of FCPs was hydrolyzed with 4 mL of 4 mol/L trifluoroacetic acid (TFA) in a sealed ampoule at 110 °C for 8 h. After hydrolysis, residual TFA was removed by repeated co-evaporation with methanol under reduced pressure five times. The dried hydrolysate was redissolved in 1 mL of deionized water for PMP derivatization. Individual monosaccharide standard solutions, including mannose, rhamnose, glucose, galactose, xylose, arabinose, ribose, glucuronic acid and galacturonic acid, were prepared at a concentration of 2.0 mg/mL. For PMP derivatization, 200 μL of sample or standard solution was mixed with 200 μL of 0.3 mol/L NaOH and 200 μL of 0.5 mol/L PMP methanol solution. The mixture was reacted at 70 °C for 1 h, cooled to room temperature, and neutralized with 200 μL of 0.3 mol/L HCl. The resulting solution was extracted three times with chloroform to remove excess PMP. The aqueous phase was collected and filtered through a 0.22 μm membrane before HPLC analysis. HPLC analysis was performed using an Agilent 1260 Infinity II Prime HPLC system equipped with a diode array detector (DAD) and an Agilent SB-C18 column (250 mm × 4.6 mm, 5 μm) (Agilent Technologies, Santa Clara, CA, USA). The mobile phase consisted of 0.1 mol/L phosphate-buffered saline (PBS) solution and acetonitrile at a ratio of 83:17 (*v*/*v*). The flow rate was 1.0 mL/min, the column temperature was maintained at 30 °C, the injection volume was 10 μL.

### 2.4. Characterization of FCPs

Fourier-transform infrared spectroscopy (FT-IR), thermal and rheological analyses were performed for supplementary physicochemical characterization of FCPs, and the detailed procedures were described in the Supplementary Materials Section S2.

### 2.5. C. elegans Strains, Bacterial Food, and Synchronization

Wild-type *C. elegans* N2 was used in this study. Worms were routinely maintained on nematode growth medium (NGM) plates seeded with *Escherichia coli* OP50 as the bacterial food source at 16 °C. For obtaining age-synchronized worms, gravid adult worms were transferred onto limiting plates without OP50 and allowed to lay eggs overnight. The adult worms were removed the next day, leaving synchronized L1 larvae on the plates. The L1 larvae were then transferred to standard OP50-seeded NGM plates and cultured until adulthood. Adult worms were used for subsequent assays.

### 2.6. FCPs Feeding Design and Lifespan Assay

FCPs treatment was started at the adult stage. To avoid differences in bacterial food availability among groups, OP50 cultures were prepared under identical conditions and adjusted to the same bacterial density before use. In the actual feeding protocol, OP50 cultures were adjusted to an OD600 of 0.6–1.0 before use, and 80 μL of OP50 suspension was added to each plate. FCPs solutions were thoroughly mixed with OP50 suspension to obtain final FCPs concentrations of 50, 100, 200, and 400 ng/mL. Considering that only very small amounts of FCPs were added in the feeding assay, the effect of the additional carbohydrate contributed by FCPs on food availability was minimal.

The control group was prepared by mixing OP50 suspension with the same volume of deionized water. For each assay, equal volumes of the OP50-FCPs mixtures or control OP50 suspension were evenly spread onto NGM plates. During treatment, worms were maintained at 16 °C and transferred to freshly prepared treatment plates every two days to maintain stable FCPs exposure.

For lifespan assays, synchronized worms were cultured on standard OP50-seeded NGM plates from the L1 stage to adulthood and then transferred to treatment plates containing OP50 supplemented with 0, 50, 100, 200, or 400 ng/mL FCPs. Each plate contained 25 worms, and three independent biological replicates were performed for each treatment. Worms that failed to respond to gentle touch with a platinum wire were scored as dead. Worms that crawled off the plate, ruptured, or underwent internal hatching were censored from the analysis. The number of live and dead worms was recorded every day.

### 2.7. Measurement of Physiological Indicators

The *C. elegans* subjected to different concentrations of FCPs for 6 d were transferred onto blank NGM plates and supplemented with 20 µL of M9 buffer. The number of body bends within a duration of 1 min was counted as a measure of locomotion. A bend refers to the movement from one direction to another followed by returning to the original direction. A total of 15 animals were analyzed for each concentration.

To estimate body volume, worms were transferred to sterile NGM plates and anesthetized using 0.1% NaN_3_. Photographs of the head, gonad opening, and tail regions of the worms were taken at the same magnification. The width and length of the worm body were measured and calculated using ImageJ 1.54p software. The formula used to calculate the volume (V) of the *C. elegans* was as follows:

R = (D_1_ + D_2_ + D_3_)/6

V = πR^2^L

where L represents the length of the *C. elegans*, R denotes its average radius, D_1_ represents the width of its head region, D_2_ represents the width of its gonad opening, and D_3_ represents the width of its tail region. A total of 15 animals were analyzed for each concentration.

To determine the brood size, *C. elegans* treated with different concentrations of FCPs were transferred to new plates every 24 h until they lost their reproductive ability. A total of 8 animals were analyzed for each concentration. All oviposition plates were cultured at 16 °C. After hatching, the number of *C. elegans* in the plate was recorded as the effective egg-laying quantity.

### 2.8. Lipofuscin Accumulation Assay

Lipofuscin accumulation was assessed based on intestinal autofluorescence in aged worms [23]. Synchronized worms were treated with FCPs at concentrations of 0, 100, and 200 ng/mL for 10 days. After treatment, worms were collected, washed with M9 buffer, and anesthetized with 0.1% NaN_3_ before being transferred onto glass slides for fluorescence imaging. Images were acquired using a fluorescence microscope (DMi8, Leica, Düsseldorf, Germany). Fluorescence images from all groups were acquired using identical microscope settings. Fluorescence intensity was quantified using ImageJ 1.54p software under the same analysis parameters for all worms. For each treatment group, 25 worms were analyzed.

### 2.9. Intracellular ROS Assay

Intracellular ROS levels were measured using H_2_DCFDA staining. Worms were incubated with 50 μM H_2_DCFDA at 25 °C for 30 min in the dark. The worms were then washed 4–5 times with M9 buffer to remove excess dye and anesthetized with 0.1% NaN_3_ for 2 min before imaging. Fluorescence images were acquired using a fluorescence microscope (DMi8, Leica, Düsseldorf, Germany). All groups within the same experiment were stained, washed, mounted, and imaged under identical conditions. Fluorescence intensity was quantified using ImageJ 1.54p software under the same analysis parameters for all worms. For each treatment group, 35 worms were analyzed.

### 2.10. Juglone Experiment

Synchronized adult *C. elegans* treated with different concentrations of FCPs for 6 d were transferred to NGM plates containing 120 µmol/L juglone. Each plate contained 25 worms and 3 parallel experiments were set for each treatment. The number of dead *C. elegans* was documented every hour for 8 h.

### 2.11. RNA Interference (RNAi)

The HT115(DE3) *Escherichia coli* strain expressing double-stranded RNA targeting the *skn-1* gene was constructed previously using the L4440 RNAi vector system (Beijing Zoman Biotechnology Co., Ltd., Beijing, China) [24]. Worms fed with HT115(DE3) transformed with the L4440 empty vector were used as controls. Lifespan assays for RNAi treated worms were performed following the previously described experimental protocol (Section 2.6).

### 2.12. Real-Time Quantitative PCR Measurement

Total RNA from *C. elegans* treated with FCPs for 6 d was obtained using Trizol reagent (Sangon Biotech Co., Ltd., Shanghai, China). RNA was reverse transcribed to cDNA using M-MLV reverse transcriptase. The RT-PCR assay was carried out using an ABI 7500/7500 real-time PCR system (Applied Biosystems, Thermo Fisher Scientific, Waltham, MA, USA). The primer sequences are listed in Table S2.

### 2.13. Statistical Analysis

All experiments were independently performed at least three times unless otherwise stated. Data are presented as mean ± SEM. Statistical analyses were performed using Origin 2021 and GraphPad Prism 8.2.1. For comparisons among multiple groups, one-way ANOVA followed by Tukey’s multiple comparison test was used. For comparisons between two groups, unpaired Student’s *t*-test was applied. Survival curves from lifespan and juglone stress assays were analyzed using the log-rank (Mantel–Cox) test. Differences were considered statistically significant at *p* < 0.05.

## 3. Results

### 3.1. Preparation and Characterization of Ficus carica Fruit Polysaccharides

FCPs were prepared from fig fruits using DES-based UAE, followed by dialysis, D101 macroporous resin purification, and lyophilization. Among the tested DES systems, ChCl/But showed the highest extraction efficiency. The optimized DES-UAE conditions were selected as follows: liquid-to-solid ratio of 20 mL/g, ultrasonic time of 50 min, water content of 10%, and extraction temperature of 60 °C (Figure S1).

**Figure 1:**
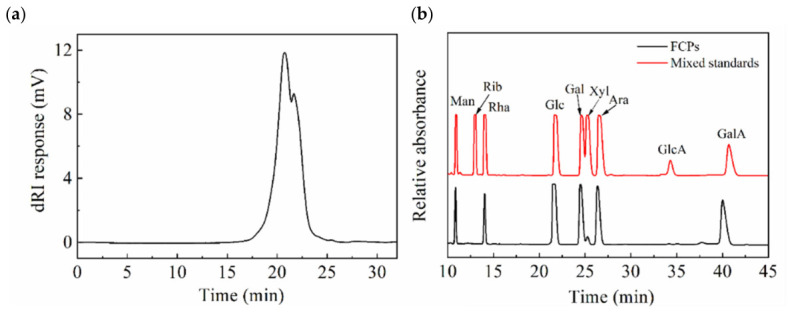
Molecular weight distribution and monosaccharide composition of FCPs. (**a**) HPGPC-dRI chromatogram of FCPs. (**b**) PMP-HPLC chromatograms of FCPs and mixed monosaccharide standards. Chromatograms in panel (**b**) were vertically offset for clarity. Man, mannose; Rib, ribose; Rha, rhamnose; Glc, glucose; Gal, galactose; Xyl, xylose; Ara, arabinose; GlcA, glucuronic acid; GalA, galacturonic acid.

The purified FCPs fraction showed a high carbohydrate content of 98.69 ± 2.42%, with a low reducing sugar content of 0.04 ± 0.01%, a negligible protein content of 0.01 ± 0.01%, and no detectable polyphenols. The uronic acid content was 21.10 ± 0.95%, indicating the presence of acidic polysaccharide components (Table S3). HPGPC analysis showed that FCPs exhibited a predominant broad elution peak with a weak shoulder at a longer retention time (Figure 1a). Based on the dextran standard calibration, the major component of FCPs had an apparent molecular weight of approximately 10.1 kDa. The broad and slightly asymmetric elution profile suggested that FCPs should be regarded as a purified fig polysaccharide fraction with a heterogeneous molecular-weight distribution [25]. Monosaccharide composition analysis showed that FCPs consisted of mannose, rhamnose, glucose, galactose, xylose, arabinose, and galacturonic acid at a molar ratio of 1.00:0.51:1.45:1.23:0.14:1.20:2.02 (Figure 1b). In addition, FT-IR (Figure S2), thermal (Figure S3), and rheological analyses (Figure S4) further confirmed the typical functional-group and physicochemical features of the polysaccharides. These results indicated that the obtained FCPs were acidic, galacturonic acid-containing polysaccharides with high carbohydrate purity, supporting their subsequent biological evaluation in *C. elegans*.

### 3.2. FCPs Extend Lifespan and Maintain Physiological Fitness in C. elegans

**Figure 2:**
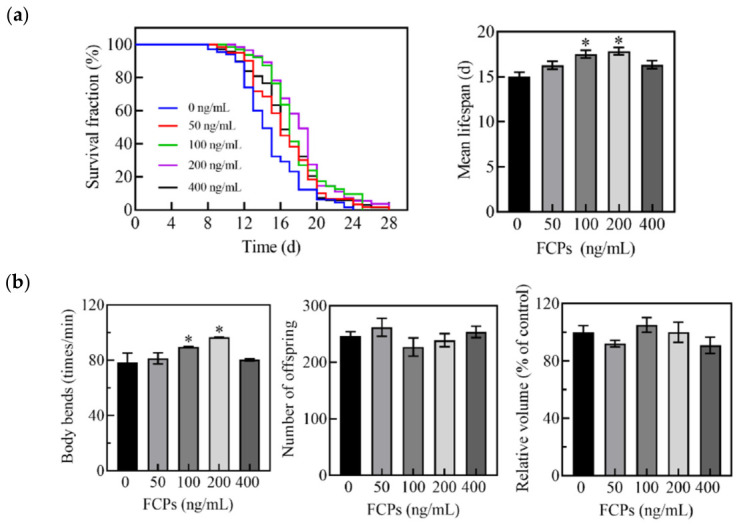
FCPs extend lifespan and maintain physiological fitness in *C. elegans*. (**a**) Survival curves and mean lifespan of worms treated with different concentrations (0, 50, 100, 200, 400 ng/mL) of FCPs. Data are representative of three independent experiments with similar results. (**b**) Physiological indicators of *C. elegans* after FCPs treatment, including body bends, number of offspring, and body volume. Body bends and body volume were assessed on day 6 of FCPs treatment, while the number of offspring was determined by counting the total offspring produced by individual worms during the reproductive period. Data are from 3 independent experiments and expressed as mean ± SEM. * *p* < 0.05 compared with the control group (0 ng/mL FCPs).

Lifespan is a key indicator of aging [26]. Therefore, the anti-aging effects of FCPs were evaluated by examining their impact on the lifespan of *C. elegans*. As shown in Figure 2a, treatment with FCPs at concentrations of 50, 100, 200, and 400 ng/mL significantly shifted the survival curves, resulting in increases in mean lifespan by 8.15%, 15.18%, 16.01%, and 7.30%, respectively. These results indicated that FCPs exerted a non-linear, hormetic-like lifespan-extending effect in *C. elegans*, with the most pronounced benefit observed at 200 ng/mL.

Extension of lifespan may sometimes be accompanied by declines in physiological fitness, such as reduced mobility, impaired reproductive capacity, or altered body size [27]. Therefore, we further evaluated whether FCPs affected healthspan-related physiological indicators in *C. elegans*. As shown in Figure 2b, treatments with 100 and 200 ng/mL FCPs significantly increased body bends, suggesting improved locomotor activity. In addition, the number of offspring was not significantly altered by FCPs treatments, indicating that the reproductive capacity was not compromised under the tested conditions. FCPs treatments did not significantly affect the body volume either, suggesting that the lifespan extension was not accompanied by obvious impairment of body size. Collectively, these results indicated that FCPs extended lifespan without compromising physiological fitness in *C. elegans*.

### 3.3. FCPs Reduce Lipofuscin Accumulation in C. elegans

**Figure 3:**
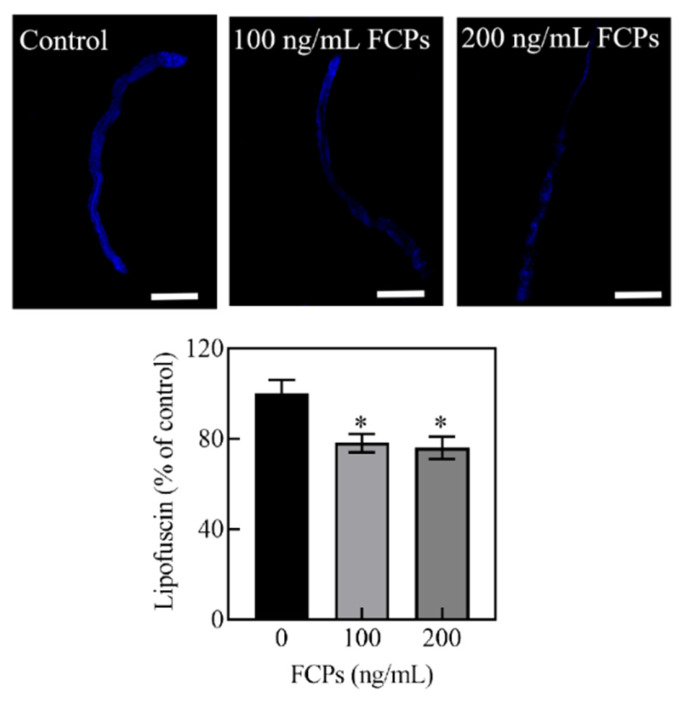
FCPs reduce lipofuscin accumulation in *C. elegans*. Representative fluorescence images of lipofuscin in worms treated with FCPs for 10 days and quantitative analysis of lipofuscin fluorescence intensity using ImageJ 1.54p. Worms were treated with 0, 100, or 200 ng/mL FCPs. Scale bar: 250 μm. Data are presented as mean ± SEM. * *p* < 0.05 compared with the control group (0 ng/mL FCPs).

Lipofuscin accumulation in the intestine of *C. elegans* is a well-established aging marker and is negatively associated with lifespan [28]. As shown in Figure 3, compared with the control group, FCPs at concentrations of 100 and 200 ng/mL significantly decreased lipofuscin fluorescence intensity by 22.91% and 24.12%, respectively (*p* < 0.05). These results indicated that FCPs reduced age-associated lipofuscin accumulation in *C. elegans*, further supporting their anti-aging effects.

### 3.4. FCPs Enhance Oxidative Stress Resistance in C. elegans

**Figure 4:**
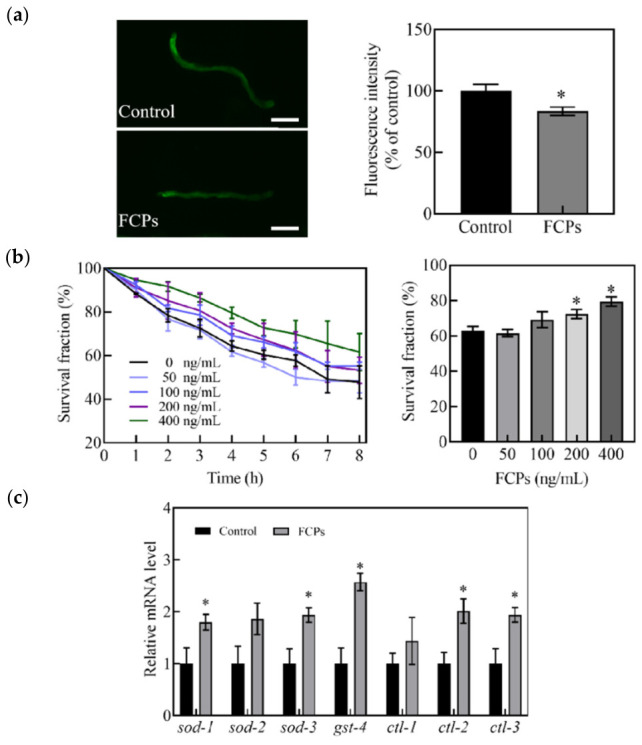
FCPs enhance oxidative stress resistance in *C. elegans*. (**a**) Representative fluorescence images and quantification of the fluorescence intensity in H_2_DCFDA-stained worms treated with 200 ng/mL FCPs for 6 days. (**b**) Survival curves of worms pretreated with different concentrations of FCPs for 6 days and then exposed to 120 μmol/L juglone-induced oxidative stress, and the survival fraction (%) at the 4 h time point. (**c**) Relative mRNA expression levels of oxidative stress-related genes in worms treated with 200 ng/mL FCPs for 6 days, as determined by qPCR and normalized to the reference gene *pmp-3*. Scale bar: 250 μm. *n* = 3, Data are presented as mean ± SEM. * *p* < 0.05 compared with the corresponding control group (Con., 0 ng/mL FCPs).

Aging is closely associated with increased ROS production and impaired oxidative stress defense, which contribute to aging-related functional decline [29]. As shown in Figure 4a, treatment with 200 ng/mL FCPs for 6 days significantly reduced ROS level by 16.4% compared with the control group, indicating that FCPs decreased intracellular oxidative stress levels. To further determine whether FCPs improve resistance to acute oxidative damage, worms pretreated with different concentrations of FCPs for 6 days were exposed to juglone-induced oxidative stress. As shown in Figure 4b, FCPs pretreatment increased the survival fraction of worms under juglone stress, with significant protective effects observed at 200 and 400 ng/mL. These results suggest that FCPs enhanced the ability of *C. elegans* to withstand oxidative stress.

We next examined the expression of oxidative stress-related genes by qPCR. In *C. elegans*, *sod-1*, *sod-2*, and *sod-3* encode different superoxide dismutases, *gst-4* encodes glutathione S-transferase, and *ctl-1*, *ctl-2*, and *ctl-3* encode different catalases; these genes are important components of the endogenous antioxidant defense system [29]. As shown in Figure 4c, treatment with 200 ng/mL FCPs for 6 days significantly upregulated the expression of *sod-1*, *sod-3*, *gst-4*, *ctl-2*, and *ctl-3* compared with the control group, whereas no significant changes were observed in *sod-2* and *ctl-1*. These results indicated that FCPs might reduce ROS level and enhance oxidative stress resistance in *C. elegans* by increasing the expression of these antioxidant defense-related genes.

### 3.5. SKN-1 Is Required for FCPs-Mediated Lifespan Extension and ROS Reduction in C. elegans

**Figure 5:**
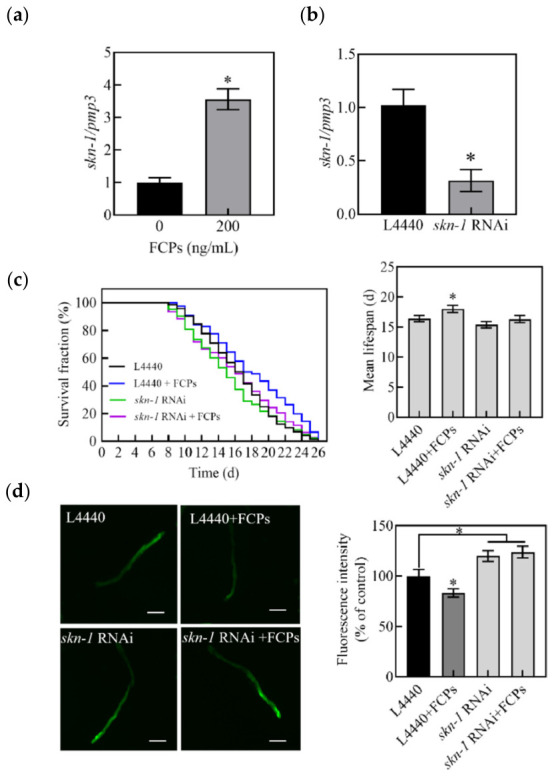
SKN-1 is required for FCPs-mediated lifespan extension and ROS reduction in *C. elegans*. (**a**) Relative mRNA expression level of *skn-1* in worms treated with or without FCPs (200 ng/mL) for 6 days, as determined by qPCR and normalized to the reference gene *pmp-3*. (**b**) Knockdown efficiency of *skn-1* RNAi. The mRNA level of *skn-1* was measured in worms fed with HT115(DE3) bacteria transformed with the L4440 empty vector or *skn-1* RNAi constructs. Relative expression levels were determined by qPCR and normalized to *pmp-3*. (**c**) Survival curves and mean lifespan of *skn-1* RNAi-treated worms following treatment with 200 ng/mL FCPs. (**d**) Representative fluorescence images and quantification of fluorescence intensity in *skn-1* RNAi-treated worms stained with H_2_DCFDA after treatment with FCPs (200 ng/mL) for 6 days. Scale bar: 100 μm. *n* = 3, Data are presented as mean ± SEM. * *p* < 0.05 compared with the corresponding control group. In panel (**a**), the corresponding control group was worms without FCPs treatment, in panels (**b**–**d**), the corresponding control group was worms fed with HT115 (DE3) bacteria transformed with the L4440 empty vector.

SKN-1, the *C. elegans* ortholog of mammalian Nrf, is a key transcription factor involved in oxidative stress responses, detoxification, and longevity regulation [30]. As shown in Figure 5a, treatment with 200 ng/mL FCPs for 6 days significantly increased *skn-1* expression compared with the control group. To further determine whether SKN-1 is required for the biological effects of FCPs, *skn-1* RNAi was performed. Before conducting lifespan and ROS assays under RNAi conditions, the knockdown efficiency of *skn-1* RNAi was verified by qPCR. As shown in Figure 5b, compared with worms fed HT115(DE3) bacteria carrying the L4440 empty vector, worms fed HT115(DE3) expressing *skn-1* RNAi constructs showed a marked reduction in *skn-1* mRNA level. The *skn-1* transcript level was reduced to approximately 30.9% of the L4440 control level, corresponding to an approximate knockdown efficiency of 69.1%. These results confirmed the effectiveness of the *skn-1* RNAi treatment used in this study.

We next examined whether FCPs could still extend lifespan when *skn-1* was knocked down. As shown in Figure 5c, FCPs treatment failed to significantly extend the lifespan of *skn-1* RNAi-treated worms. This result indicated that the lifespan-promoting effect of FCPs was largely attenuated upon *skn-1* knockdown, suggesting that SKN-1 was required for FCPs-mediated lifespan extension.

ROS levels were then examined in *skn-1* RNAi-treated worms. As shown in Figure 5d, FCPs treatment did not significantly reduce ROS level in *skn-1* RNAi-treated worms compared with the corresponding control group. These results suggested that the ROS-reducing effect of FCPs also depended on SKN-1. Collectively, these findings indicated that SKN-1 was required for the lifespan-extending and oxidative stress-reducing effects of FCPs in *C. elegans*.

### 3.6. FCPs Are Associated with Altered Expression of Selected IIS Pathway-Related Genes in C. elegans

The insulin/IGF-1 signaling (IIS) pathway is a conserved aging-related pathway and has been reported to interact with SKN-1-mediated stress-response regulation in *C. elegans* [31]. In the canonical IIS cascade, *daf-2* encodes the insulin/IGF-1 receptor-like protein, *age-1* encodes a phosphoinositide 3-kinase, *akt-1* and *akt-2* encode AKT/protein kinase B homologs, and *daf-16* encodes the FOXO transcription factor [32,33,34].

**Figure 6:**
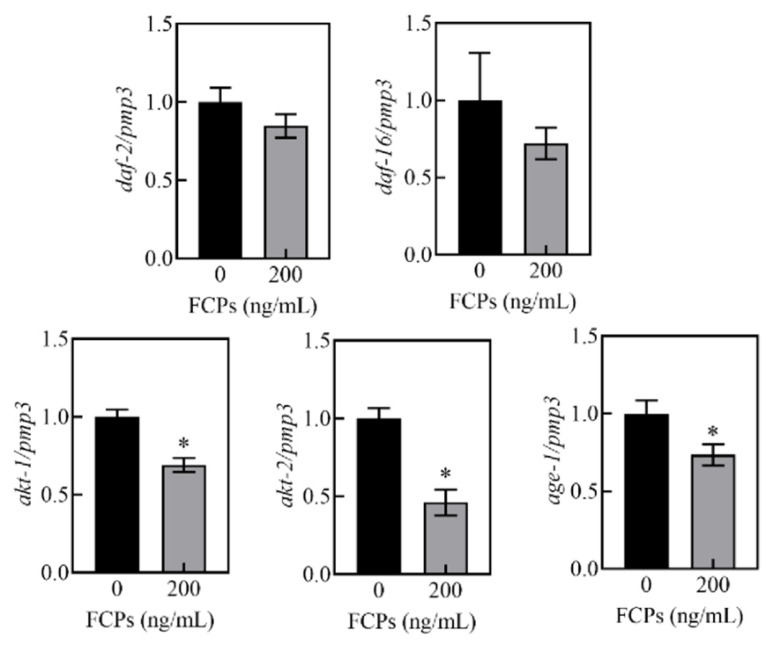
FCPs are associated with altered expression of selected IIS pathway-related genes in *C. elegans*. Worms were treated with or without FCPs (200 ng/mL) for 6 days prior to RNA extraction. Relative mRNA expression levels of selected IIS pathway-related genes, including *daf-2*, *age-1*, *akt-1*, *akt-2*, and *daf-16*, were determined by qPCR and normalized to the reference gene *pmp-3*. *n* = 3, Data are presented as mean ± SEM. * *p* < 0.05 compared with the corresponding control group (0 ng/mL FCPs).

As shown in Figure 6, treatment with 200 ng/mL FCPs for 6 days significantly downregulated the expression of *age-1*, *akt-1*, and *akt-2* compared with the control group (*p* < 0.05), whereas no significant changes were observed in the expression levels of *daf-2* and *daf-16*. These results suggested that FCPs treatment was associated with altered expression of selected IIS pathway-related genes, particularly *age-1*, *akt-1*, and *akt-2*.

## 4. Discussion

Natural polysaccharides have attracted increasing attention as promising anti-aging agents due to their structural diversity, low toxicity, and suitability for long-term dietary intake [35]. Accumulating evidence indicates that plant-derived polysaccharides can delay aging-associated functional decline by modulating stress resistance and cellular homeostasis [36,37,38,39]. Although previous studies have reported the extraction, characterization, and bioactivities of natural polysaccharides [17,40,41], the mechanistic basis by which polysaccharides regulate organismal lifespan, particularly through conserved aging pathways in vivo, remains insufficiently defined. In this context, the present study evaluated the lifespan-extending effects of a fig fruit polysaccharide fraction, FCPs, in *C. elegans* and investigated its association with oxidative stress resistance and SKN-1 expression.

The preliminary physicochemical characterization showed that FCPs contained a major polysaccharide peak with an apparent molecular weight of approximately 10,118 Da and a minor low-molecular-weight peak, and were composed of multiple monosaccharides, including galacturonic acid, arabinose, galactose, glucose, mannose, rhamnose, and xylose. Previous studies have suggested that the anti-aging potential of polysaccharides is closely associated with their structural features, including molecular weight distribution, monosaccharide composition, and uronic acid content [37,42]. Acidic polysaccharides enriched in uronic acids and neutral sugar residues are frequently reported to exhibit antioxidant and stress-regulatory activities, possibly due to their solubility, charge properties, and interactions with cellular signaling components [42]. Therefore, the compositional features of FCPs may partly contribute to their biological activity.

In *C. elegans* model, FCPs significantly extended the lifespan while maintaining physiological fitness, as indicated by unchanged body volume and offspring number. Notably, as the concentration of FCPs increased, their lifespan-promoting effect first increased and then decreased, with the most pronounced effect observed at 200 ng/mL. Such a response is consistent with the concept of hormesis, in which moderate stimulation can activate adaptive protective mechanisms, whereas excessive exposure does not further enhance the beneficial effect [26]. In this context, FCPs may function as mild nutritional or signaling modulators that promote longevity within an appropriate concentration range.

The oxidative stress-related results further support this interpretation. FCPs reduced ROS level, improved survival under juglone-induced oxidative stress, and upregulated several antioxidant defense-related genes, including *sod-1*, *sod-3*, *gst-4*, *ctl-2*, and *ctl-3*. In *C. elegans*, oxidative stress resistance is not determined solely by direct radical scavenging but is strongly influenced by endogenous stress-response pathways and transcriptional regulation [29,30]. Therefore, the reduced ROS level observed in FCPs-treated worms may reflect enhanced endogenous defense capacity rather than only a direct antioxidant effect. This interpretation is also consistent with the upregulation of genes encoding superoxide dismutases, glutathione S-transferase, and catalases in FCPs-treated worms, which are important components of the antioxidant defense system [29,43].

Among the transcriptional regulators involved in stress adaptation, SKN-1, the *C. elegans* ortholog of mammalian Nrf, plays a central role in oxidative stress defense, detoxification, and longevity regulation [7,30]. In this study, FCPs increased the expression of *skn-1*. More importantly, the lifespan-extending effect of FCPs was lost in *skn-1* RNAi-treated worms, and FCPs failed to significantly reduce ROS level under *skn-1* knockdown conditions. These results provided evidence that SKN-1 was required for the longevity-promoting and oxidative stress-reducing effects of FCPs. Thus, FCPs appeared to promote longevity through an SKN-1-dependent stress-defense response rather than through direct ROS scavenging alone. In addition, FCPs treatment was accompanied by altered expression of selected IIS pathway-related genes, including *age-1*, *akt-1*, and *akt-2*, suggesting that changes in IIS pathway might also be involved in the response to FCPs.

Taken together, the present study demonstrated that FCPs promoted longevity and oxidative stress resistance in *C. elegans* through a mechanism dependent on SKN-1 and accompanied by altered expression of selected IIS-related genes.

Future studies should further separate FCPs into defined subfractions, clarify their fine structural features, and compare the biological activities of different fractions. In addition, further genetic and reporter-based assays are needed to clarify the relationship between IIS-related transcriptional changes and SKN-1-dependent stress-defense responses.

## 5. Conclusions

In summary, a fig-derived polysaccharide fraction, FCPs, was obtained from *Ficus carica* L. fruits using DES-based UAE. The obtained FCPs extended lifespan while improving locomotor activity without affecting the body size or reproductive capacity in *C. elegans*. The longevity-promoting and ROS-reducing effects of FCPs depended on SKN-1. This study provides evidence that green-extracted fig polysaccharides promote longevity and oxidative stress resistance in *C. elegans* through an SKN-1-dependent mechanism. Further studies in more complex animal models are needed before extrapolating these findings to mammals or humans.

## Data Availability

Data are contained within the article and Supplementary Materials.

## Supplementary Materials

The following supporting information can be downloaded at: https://www.mdpi.com/article/10.3390/antiox15060691/s1. References [44,45,46,47,48,49,50,51,52] are cited in the Supplementary Materials.

## Abbreviations

The following abbreviations are used in this manuscript:

DES	deep eutectic solvents
UAE	ultrasound-assisted extraction
DES-UAE	deep eutectic solvent-based ultrasound-assisted extraction
HBA	hydrogen bond acceptor
HBD	hydrogen bond donor
ChCl	choline chloride
ChCl/Urea	choline chloride/urea
ChCl/Lac	choline chloride/lactic acid
ChCl/Oxa	choline chloride/oxalic acid
Gly/Gla/H_2_O	glycerin/glucose/water
Gly/Thr/H_2_O	glycerin/threonine/water
Ace/Lac	acetamide/lactic acid
ChCl/EGly	choline chloride/glycol
ChCl/But	choline chloride/1,3-butanediol
ChCl/D-Sor	choline chloride/sorbitol
ChCl/But/D-Sor	choline chloride/1,3-butanediol/sorbitol
FT-IR	Fourier-transform infrared spectroscopy
TGA	thermogravimetric analysis
HPGPC-dRI	high-performance gel permeation chromatography coupled with differential refractive index detection
HPLC	high-performance liquid chromatography
PMP	1-phenyl-3-methyl-5-pyrazolone
DAD	diode array detector
TFA	trifluoroacetic acid
RNAi	RNA interference
qPCR	quantitative real-time PCR
cDNA	complementary DNA
ROS	reactive oxygen species
H_2_DCFDA	2′,7′-dichlorodihydrofluorescein diacetate
IIS	insulin/IGF-1 signaling
IGF-1	insulin-like growth factor 1
SKN-1	skinhead-1
Nrf	nuclear factor erythroid 2-related factor
DAF-16	abnormal dauer formation protein 16
FOXO	forkhead box O
SOD	superoxide dismutase
GST	glutathione S-transferase
CTL	catalase
