# Supplementary material for: Green-Extracted Ficus carica L. Fruit Polysaccharides Promote Longevity in Caenorhabditis elegans via Modulation of SKN-1 and IIS Pathway
Source: Antioxidants (Basel). 2026 May 30;15(6):691. doi: 10.3390/antiox15060691 (PMC13296142; doi:10.3390/antiox15060691)
Supplement: Supplementary file 1 [file antioxidants-15-00691-s001.zip › antioxidants-4295320-supplementary.pdf]

Supplementary Methods

S1. Preparation of DESs and UAE-DES extraction optimization

DESs were prepared by mixing the corresponding hydrogen bond acceptor (HBA) and hydrogen bond donor (HBD) components at the designed molar ratios. The detailed compositions, molar ratios, reaction temperatures, and reaction times of the DES systems are listed in Table S1. Briefly, the components were weighed, mixed in a sealed glass vessel, and heated under continuous magnetic stirring until a clear and homogeneous liquid was obtained.

For DES screening, 1.0 g of pretreated *Ficus carica* fruit powder was mixed with 10.0 mL of each DES system. Extraction was performed at 60 °C for 30 min with shaking at 100 rpm. Deionized water was used as the conventional extraction solvent for comparison. After extraction, the mixture was centrifuged at 4000 × g for 15 min, and the supernatant was collected for polysaccharide determination using the phenol-sulfuric acid method.

After ChCl/But was selected as the extraction solvent according to polysaccharide yield, single-factor experiments were performed to optimize the UAE-DES extraction conditions. In each single-factor experiment, only one parameter was varied, while the other extraction conditions were kept constant. The ultrasonic power was fixed at 600 W throughout the optimization experiments.

First, the liquid-to-solid ratio was optimized by testing ratios of 10, 20, 30, 40, and 50 mL/g, while ChCl/But containing 10% (v/v) water was used as the extraction solvent, and the ultrasonic time and extraction temperature were fixed at 30 min and 60 °C, respectively. Based on this experiment, 20 mL/g was selected for subsequent optimization. Second, the ultrasonic time was optimized by testing extraction times of 20, 30, 40, 50, and 60 min. During this step, the liquid-to-solid ratio was fixed at 20 mL/g, ChCl/But containing 10% (v/v) water was used as the extraction solvent, and the extraction temperature was fixed at 60 °C. Based on this experiment, 50 min was selected for subsequent optimization. Third, the water content of ChCl/But was optimized by testing water contents of 0%, 5%, 10%, 15%, and 20% (v/v). During this step, the liquid-to-solid ratio, ultrasonic time, and extraction temperature were fixed at 20 mL/g, 50 min, and 60 °C, respectively. Based on this experiment, 10% (v/v) water content was selected for subsequent extraction. Finally, the extraction temperature was optimized by testing temperatures of 30, 40, 50, 60, and 70 °C. During this step, ChCl/But containing 10% (v/v) water was used as the extraction solvent, and the liquid-to-solid ratio and ultrasonic time were fixed at 20 mL/g and 50 min, respectively. Based on this experiment, 60 °C was selected as the optimal extraction temperature.

After each extraction, the mixture was centrifuged at 4000 × g for 15 min. The polysaccharide concentration in the supernatant was determined using the phenol-sulfuric acid method with glucose as the standard. The polysaccharide yield was calculated according to the following equation:

$$Y\ (\%) = C \times V / W \times 100$$

Where Y is the polysaccharide yield (%), C is the polysaccharide concentration in the extract (mg/mL), V is the volume of the extract (mL), and W is the mass of the pretreated fig fruit powder (mg).

Based on the single-factor optimization results, the final UAE-DES extraction conditions were selected as follows: liquid-to-solid ratio of 20 mL/g, ultrasonic power of 600 W, ultrasonic time of 50 min, water content of 10% (v/v), and extraction temperature of 60 °C. These optimized conditions were used for the preparative extraction of FCPs.

S2. Supplementary physicochemical characterization of FCPs

Supplementary physicochemical characterization was performed to further determine the functional groups, thermal stability, and rheological properties of the purified FCPs fraction.

The functional groups of FCPs were analyzed by Fourier-transform infrared spectroscopy (FT-IR). Freeze-dried FCPs powder was thoroughly mixed with dried KBr at a mass ratio of 1:100 and pressed into a transparent pellet. The FT-IR spectrum was recorded using an FT-IR spectrometer (Tensor 27, Bruker, Germany) over the wavenumber range of 4000–400 cm^{-1}.

Thermal stability was evaluated using thermogravimetric analysis (TG) and derivative thermogravimetry (DTG). Freeze-dried FCPs powder was placed in an alumina crucible and analyzed using a simultaneous thermal analyzer (STA449F5, Netzsch GmbH, Germany). The sample was heated from 30 °C to 600 °C at a heating rate of 10 °C/min, and the TG and DTG curves were recorded.

The rheological properties of FCPs solution were measured using a rheometer (TA Instruments, New Castle, USA) equipped with a cone-plate geometry with a plate diameter of 60 mm, cone angle of 1°, and gap of 1 mm. Before measurement, FCPs were dissolved in deionized water to obtain a homogeneous polysaccharide solution. Dynamic frequency sweep measurements were conducted to determine the storage modulus (G′) and loss modulus (G″) as functions of angular frequency. Steady shear measurements were performed to evaluate the apparent viscosity of FCPs as a function of shear rate.

Supplementary Tables

Table S1. Synthesis conditions for 10 types of DESs

HBA/HBD	Molar ratio	Temperature (°C)	Reaction time (h)
ChCl/Urea	1:2	80	2.0
ChCl/Lac	1:1	80	2.5
ChCl/Oxa	1:1	80	3.5
Gly/Gla/H_2O	3:1:1	90	2.5
Gly/Thr/H_2O	3:1:1	90	2.5
Ace/Lac	1:1	80	2.5
ChCl/EGly	1:2	80	2.0
ChCl/But	1:2	80	1.0
ChCl/D-Sor	1:2	90	2.5
ChCl/But/D-Sor	1:1:1	90	3.0

Table S2. Gene primer sequences

Gene	Forward Primer (5'→3')	Reverse Primer (5'→3')
pmp-3	GTTCCCGTGTTCATCACTCAT	ACACCGTCGAGAAGCTGTAGA
sod-1	ACGCTTTACGGTCCAAACACT	CTTGGACTCTTCTGCCTTGTCT
sod-2	AAACAGCTTTCGGCATCAAC	TTCCGAACAGTGGAACAAGTC
sod-3	AGAACCTTCAAAGGAGCTGATG	CCGCAATAGTGATGTCAGAAAG
ctl-1	TGATCGAGGTCGCCAAGATG	CAAGGCGATGGTAATGCGTG
ctl-2	TCCGTGACCCTATCCACTTC	TGGGATCCGTATCCATTCAT
ctl-3	TGATCGAGGTCGGCAAGATG	CAAGGCGATGGTAATGCGTG
gst-4	TTCGGTGGTTCCTTTGGTG	GGAGAAGAAGCAGAGGGAGA
daf-2	TCAAATGAACGAGGAGCCG	TGGAACACCGTAGGAAGAGC
daf-16	TCGTCGTCTCGTGTTTCTCCA	TTCCATAGGCACCCGGTAGTG
akt-1	CAAAGCCTAAGGAAGGACAACC	TTCCATAGGCACCCGGTAGTG
akt-2	ACATTCAGCGAAGCACGAACA	CATGAATCCAACGCTGACGAAC
age-1	CGGAAAGACCAAACTTGGGAT	CGTAGGCTTCGACGCATAACG
skn-1	ACGCCAATTTGCTCCAGTCT	GATGGCAAACTTTTGATCATTGTTA

Table S3. Chemical composition of FCPs

Parameter	Content (%)
Total sugar content	98.69 ± 2.42
Reducing sugar content	0.04 ± 0.01
Protein content	0.01 ± 0.01
Polyphenol content	N.d.
Uronic acid content	21.1 ± 0.95

Note: Values are expressed as mean ± SEM. N.d., not detected.

Supplementary Figures

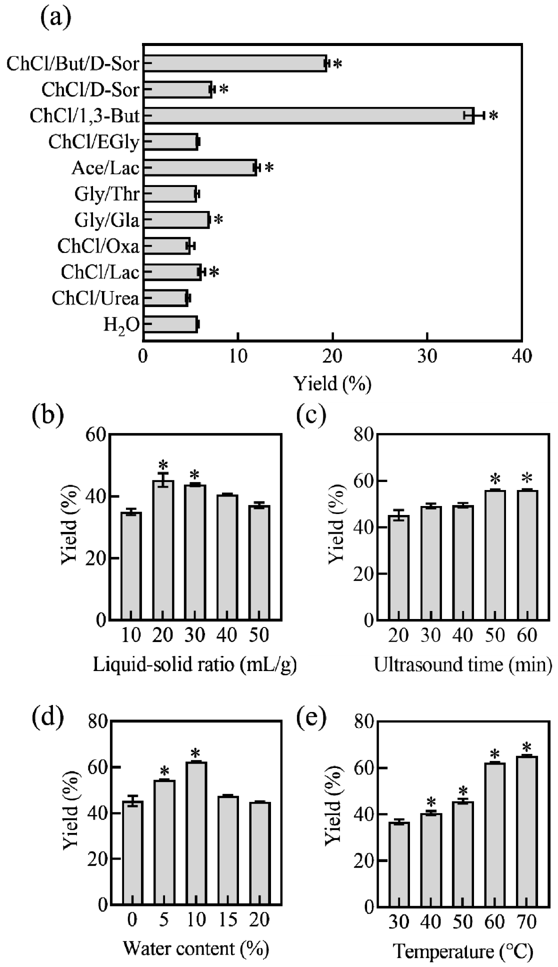

Figure. S1. Effects of UAE-DES extraction conditions on the polysaccharide yield of FCPs.

(a) Effect of different extraction solvents. (b) Effect of liquid-to-solid ratio. (c) Effect of ultrasound time. (d) Effect of water content. (e) Effect of extraction temperature. ChCl/But showed the highest polysaccharide yield among the tested DES systems and was selected for subsequent single-factor optimization. Based on the optimization results, the final UAE-DES extraction conditions were selected as follows: liquid-to-solid ratio, 20 mL/g; ultrasound power, 600 W; ultrasound time, 50 min; water content, 10% (v/v); and extraction temperature, 60 °C. Detailed fixed conditions for each single-factor experiment are provided in Supplementary Methods S1, and the full names of the DES systems are listed in the Abbreviations section. Data are presented as mean ± SEM. In panel (a), $p < 0.05$ compared with the water extraction group. In panel (b), $p < 0.05$ compared with the 10 mL/g group. In panel (c), $p < 0.05$ compared with the 20 min group. In panel (d), $p < 0.05$ compared with the 0% water content group. In panel (e), $p < 0.05$ compared with the 30 °C group.

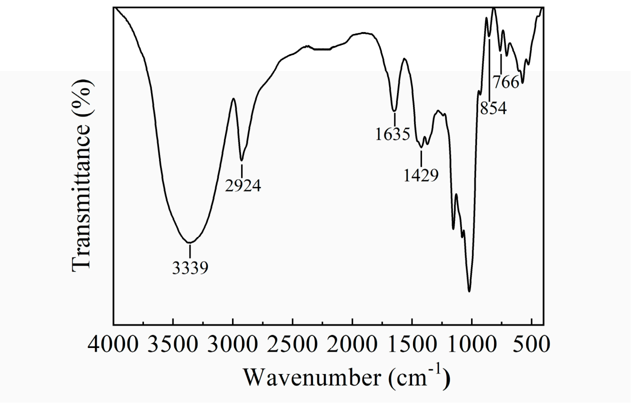

Figure. S2. FT-IR spectrum of FCPs.

The broad absorption around 3339 cm⁻¹ and the band near 2924 cm⁻¹ were assigned to O–H and C–H stretching vibrations, respectively [44,45]. The absorption bands around 1635 and 1429 cm⁻¹ suggested the presence of carboxyl-related groups, consistent with the uronic acid-containing nature of FCPs [44,46]. The absorptions in the 1200–1000 cm⁻¹ region were characteristic of glycosidic bond-related vibrations in polysaccharides [47]. These results support the typical FT-IR features of polysaccharides in the purified FCPs fraction.

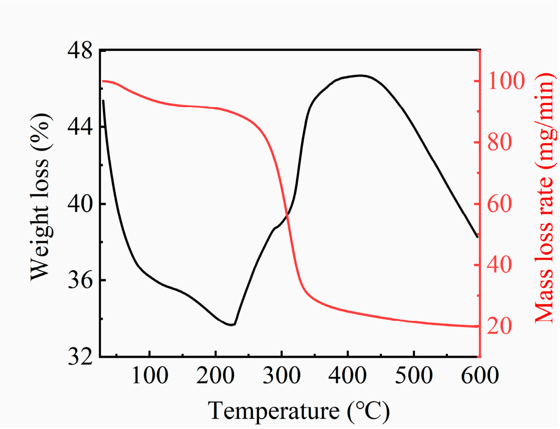

Figure. S3. Thermal stability of FCPs.

The TG/DTG curves showed a three-stage weight-loss pattern. The initial weight loss at 30–210 °C was mainly attributed to the evaporation of free and bound water [48]. The major weight loss at 210–320 °C was associated with thermal degradation of the polysaccharide matrix, including cleavage of glycosidic linkages and decomposition of carbohydrate chains [49]. The slow weight loss at 320–600 °C reflected further decomposition and carbonization of residual components [50].

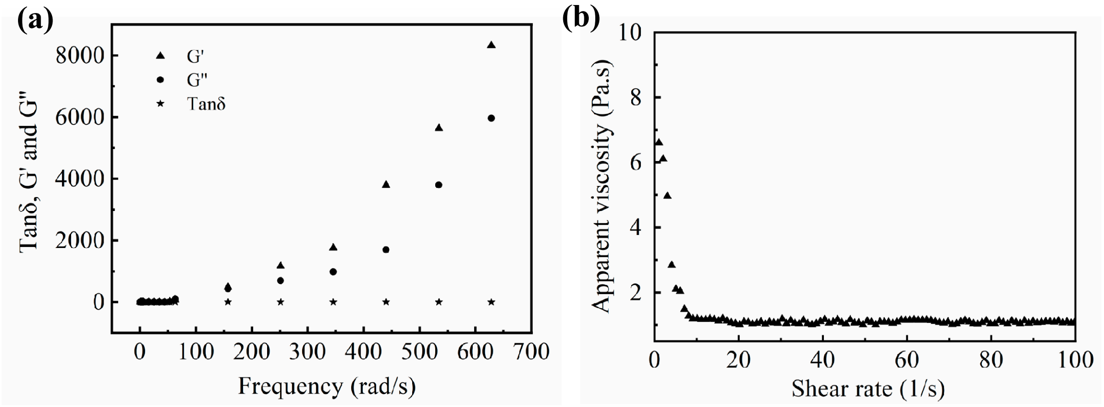

Figure. S4. Rheological properties of FCPs.

(a) Dynamic viscoelastic properties (storage modulus G' and loss modulus G") as a function of angular frequency. (b) Steady-state shear viscosity as a function of shear rate. Rheological analysis showed that both storage modulus (G′) and loss modulus (G″) increased with increasing angular frequency, indicating frequency-dependent viscoelastic behavior of the FCPs solution [51]. In addition, the apparent viscosity decreased with increasing shear rate, suggesting that FCPs exhibited shear-thinning behavior, which is typical of pseudoplastic polysaccharide solutions[52]
